# Cloning and characterization of canine *PAX6* and evaluation as a candidate gene in a canine model of aniridia

**Published:** 2007-03-26

**Authors:** Linda S. Hunter, Duska J. Sidjanin, Manuel Villagrasa Hijar, Jennifer L. Johnson, Ewen Kirkness, Gregory M. Acland, Gustavo D. Aguirre

**Affiliations:** 1 J.A. Baker Institute for Animal Health, College of Veterinary Medicine, Cornell University, Ithaca, NY; 2 Department of Ophthalmology, The Medical College of Wisconsin, Milwaukee WI; 3 The Veterinary Ophthalmology Center, Madrid, Spain; 4 The Institute for Genomic Research, Rockville, MD; 5 Department of Clinical Studies, School of Veterinary Medicine, University of Pennsylvania, Philadelphia, PA

## Abstract

**Purpose:**

Mutations in *PAX6* cause human aniridia. The *small eye* (*sey*) mouse represents an animal model for aniridia. However, no large animal model currently exists. We cloned and characterized canine *PAX6*, and evaluated *PAX6* for causal associations with inherited aniridia in dogs.

**Methods:**

Canine *PAX6* was cloned from a canine retinal cDNA library using primers designed from human and mouse *PAX6* consensus sequences. An RH3000 radiation hybrid panel was used to localize *PAX6* within the canine genome. Genomic DNA was extracted from whole blood of dogs with inherited aniridia, and association testing was performed using markers on CFA18. Fourteen *PAX6* exons were sequenced and scanned for mutations, and a Southern blot was used to test for large deletions.

**Results:**

Like the human gene, canine *PAX6* has 13 exons and 12 introns, plus an alternatively spliced exon (5a). *PAX6* nucleotide and amino acid sequences were highly conserved between dog, human, and mouse. The canine *PAX6* cDNA sequence determined in this study spans 2 large gaps present in the current canine genomic sequence. Radiation hybrid mapping placed canine *PAX6* on CFA18 in a region with synteny to HSA11p13. Exon-scanning revealed single nucleotide polymorphisms, but no pathological mutations, and Southern blot analysis revealed no differences between normal and affected animals.

**Conclusions:**

Canine *PAX6* was cloned and characterized, and results provide sequence information for gaps in the current canine genome sequence. Canine *PAX6* nucleotide and amino acid sequences, as well as gene organization and map location, were highly homologous with that of the human gene. *PAX6* was evaluated in dogs with an inherited form of aniridia, and sequence analysis indicated no pathological mutations in the coding regions or splice sites of aniridia-affected dogs, and Southern blot analysis showed no large deletions.

## Introduction

Mutations in the paired-box 6 (*PAX6*) gene cause aniridia in man, a panocular disorder primarily characterized by complete or partial absence of iris tissue. Aniridia is often associated with other ocular abnormalities including corneal dystrophy, cataract, and glaucoma. The disease has a high penetrance, but variable expressivity, and, within pedigrees, phenotypes can vary in patients having the same mutation [1]. The incidence of aniridia is 1:65,000-1:100,000 in man, and approximately 2/3 of aniridia cases are inherited. In addition to aniridia, *PAX6* mutations (OMIM 607108) can also cause Peter's anomaly, cataract, corneal dystrophy, keratitis, coloboma, ectopic pupillae, foveal hypoplasia, and optic nerve hypoplasia. Over 300 *PAX6* mutations have been described in man, and can be viewed at the Human *PAX6* Allelic Variant Database. *PAX6* mutations are dominant to semidominant and homozygous lethal. Homozygous or compound heterozygous patients have anophthalmia and multiple, severe CNS malformations [2,3].

*PAX6* is expressed during embryogenesis in the developing CNS, eye, nose, pituitary, and pancreas [4-8]. It functions as a master control gene in ocular development across species, both vertebrate and invertebrate, and is able to induce ectopic eye development in *Drosophila melanogaster* [9] and *Xenopus laevis* [10]. Mutations in the *Drosophila PAX6* homolog *eyeless* cause partial to complete loss of the compound eye, and surrounding sensory bristles [11]. *Pax6* mutations in mouse are dominant, and result in the *small eye* (*sey*) phenotype characterized by microphthalmia and small body size [12]. Experimentally induced mutations in mouse *Pax6* exhibit a range of phenotypes, including partial to complete aniridia, iris abnormalities, cataract, and corneal adhesions [13]. Homozygous *Pax6* mutations are lethal in the mouse, and, as in man, result in small embryos with anopthalmia and severe CNS malformations [3,12].

*PAX6* is a highly conserved member of the PAX family of DNA-binding transcription factors. It contains a paired domain (PD), a linker region, a homeodomain (HD), and a proline-serine-threonine rich (PST) region which functions as a transcription activator [3]. Both the paired and homeo domains of the PAX6 protein recognize and bind to DNA target sequences. The paired domain is further divided into an NH_2_-terminal subdomain (NTS) and a COOH-terminal subdomain (CTS). An alternatively spliced form of PAX6 inserts an additional 14 amino acids, encoded by exon 5a, into the NTS of the paired domain, thereby altering its binding ability [14,15]. In the shorter PAX6, the NTS binds to DNA targets while in the longer PAX6(5a) the CTS preferentially binds. Thus, the NTS and the CTS apparently negatively regulate each other allowing the alternatively spliced exon 5a to act as a molecular switch which can determine DNA-binding targets [14,16].

In an effort to identify the causal gene, and mutation responsible for canine aniridia, a family of Spanish Catalan sheepdogs with an inherited form of aniridia was analyzed. Affected dogs presented with partial to nearly complete absence of the iris with or without keratitis, cataract, and glaucoma. Because limited pedigree information and samples precluded a genome wide scan, a candidate gene approach was used. *PAX6* was selected for evaluation because it is the primary aniridia gene in man. The canine *PAX6* has not been cloned, and the current draft of the canine genomic sequence [17] does not contain an annotated *PAX6*. Moreover, the canine draft genomic sequence does not contain the entire sequence for this gene. As a result, *PAX6* was cloned from a canine retinal cDNA library, characterized, and evaluated for mutations that could be causally associated with the disease.

## Methods

### Animals and DNA

**Figure 1:**
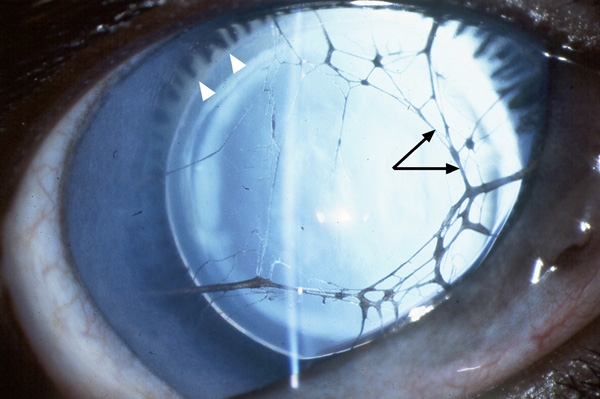
The canine aniridia phenotype. Note the absence of iris, visible ciliary processes located near the lens equator (white arrowheads) and pupillary membrane remnants (black arrows).

**Figure 2:**
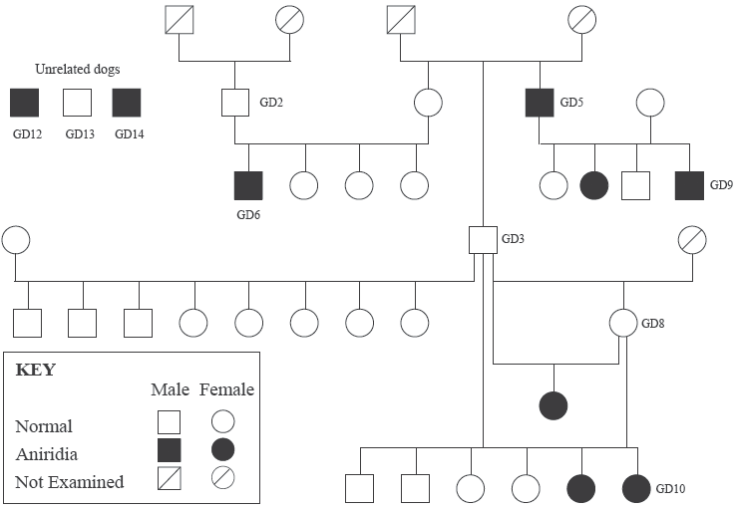
Spanish Catalan sheepdog pedigree showing aniridia-affected dogs. Note that the parents of affected dogs in 3 of the matings were clinically normal.

Catalan sheepdogs (Gos d'Atura) with an inherited form of aniridia were ascertained by Dr. Manuel Villagrasa at the Veterinary Ophthalmology Center in Madrid, Spain [18]. The dogs were privately owned pets or working sheepedogs, and were not part of a research colony. Dogs were examined using direct and indirect ophthalmoscopy and slit lamp biomicroscopy. Clinical signs included partial to nearly complete absence of the iris with ciliary processes visible at the circumferential border (Figure 1). Some canine patients also exhibited persistent remnants from the pupillary membrane, corneal edema, keratitis, cataract, and glaucoma. A total of 10 dogs were examined, including 7 which were part of one pedigree (GD2, GD3, GD5, GD6, GD8, GD9, and GD10; Figure 2), and 3 which were unrelated (GD12, GD13, and GD14). Six dogs were affected with aniridia (GD5, GD6, GD9, GD10, GD12, and GD14), and 4 were normal (GD2, GD3, GD8, and GD13). Although the pedigree information available was very limited, the disease appeared to segregate as an autosomal recessive trait in that aniridia-affected dogs were produced from phenotypically normal parents (Figure 2) [18]. Whole blood was collected in EDTA-anticoagulant tubes, and total genomic DNA (gDNA) was extracted from blood lymphocytes using standard phenol:chloroform extraction protocols with ethanol precipitation [19].

### Cloning and characterization of canine *PAX6*

Oligonucleotide primers were designed based on human (XM_012065) and mouse (X63963) *PAX6* consensus sequences using Amplify© software (Bill Engels, University of Wisconsin, Madison, WI). *PAX6* was amplified from a canine retinal cDNA library made with the pBK-CMV phagemid vector (Stratagene, LaJolla, CA), using primers listed in Table 1. Vector primers PBKIII and PBKVI from the cDNA library were used to extend the resulting *PAX6* sequence in the 5' and 3' directions, respectively. Additional sequence information was obtained from The Institute for Genomic Research (TIGR) 1.5X (poodle) canine genomic sequence [20]. Additional primers were designed to cross introns in gDNA in order to obtain the intron/exon boundary sequences which were then used to design primers for exon scanning. Intron/exon boundaries were analyzed, and intronic sizes were determined for 9 of the 13 introns. For the larger introns (2, 4, 7, and 11), which could not be amplified directly, genomic sequence at the intron/exon boundaries was obtained from TIGR (poodle) canine genomic sequence [20], and used to design primers for exon scanning.

**Table 1:** Primer sequences used to clone *PAX6* from a canine retinal cDNA library.

Primer	Location	Sequence	Tm (°C)	Product size (bp)
PAX6-1F	exons 4-5	GCAGAACAGTCACAGCGGAGTG	60.4	464
PAX6-3R	exon 7	CCGTCTGCGCCCATCTGTTG	61.4	
PAX6-4F	exon 7	GTCATCAATAAACAGAGTTCTTCGC	54.3	485
PAX6-5R	exon 10	GTGTTGCTGGCCTGTCTTCTCTG	60.1	
PAX6-6F	exon 9	GATCTACCTGAAGCAAGAATACAGG	54.9	380
PAX6-7R	exon 12	GGTGTAGGTATCATAACTCCG	51.9	
Primers used to clone 5' end of PAX6				
PBKIII F	vector	GGTCGACACTAGTGGATCCAAAG	57.1	550
PAX6-3R	exon 7	CCGTCTGCGCCCATCTGTTG	61.4	
Primers used to clone 3' end of PAX6				
PAX6-6F	exon 9	GATCTACCTGAAGCAAGAATACAGG	54.9	600
PBKVI R	vector	GCTCTCATGAAGATCTCGCCG	57.7	
PAX6-27F	exon 12	AACAGTCAGCCAATGGGCAC	53.7	200
PBKVI R	vector	GCTCTCATGAAGATCTCGCCG	57.7	
Primers used to amplify alternatively transcribed EXON 5a				
PAX6-14F	exon 5	CTCGGTGGTGTCTTTGTCAAC	54.2	250
PAX6-15R	exon 6	CTACTCTCGGTTTACTACCACC	54.7	

PCRs for the cloning of *PAX6* were run in a 25 μl reaction volume with the following conditions: 2 min at 94 °C followed by 30 cycles of 60 s at 94 °C, 60 s at 58 °C, 60 s at 72 °C, and a final extension of 7 min at 72 °C. PCRs were run on Amplitron II® Thermolyne thermocyclers (Barnstead Thermolyne Co., Dubuque, IA), and amplified products were electrophoresed on 2% agarose gels (Invitrogen, Carlsbad, CA) with 1X TAE buffer (0.04 M Tris-acetate buffer, pH 8, 100 mM EDTA) at 60V. Gels were stained with ethidium bromide, visualized using an ultraviolet transilluminator (Spectronics Corporation, Westbury, NY), and digitally photographed. PCR products were purified using the Concert^TM^ PCR purification kit (Gibco, BRL, Gaithersburg, MD), or gel bands were excised and purified using the Qiaex II® Gel Extraction kit (Qiagen Inc., Valencia, CA). Purified PCR products were then sequenced on the Applied Biosystems Automated 3730 DNA Analyzer using Big Dye Terminator chemistry and Ampli Taq-FS DNA Polymerase. Sequence assembly and analysis were performed using Sequencher^TM^ software (Gene Codes Corporation, Ann Arbor, MI).

### Exon scanning

*PAX6* exons, along with their intron/exon junctions, were scanned using PCR amplification of gDNA from 4 aniridia-affected dogs (GD5, GD6, GD9, and GD10), and 3 non-affected dogs (GD2, GD3, and GD8) from the same pedigree. In addition, one non-affected unrelated Beagle (B15) was scanned as a control. Primers were designed in introns near the intron/exon boundaries (Table 2) using Amplify software. Primers in nearby exons were used in the amplification of exons 5a, 6, 9, 10, and 11. PCR and electrophoresis conditions were the same as those described earlier. Gel bands corresponding to exon sizes were excised and gel purified using the QIAquick® Gel Extraction kit (Qiagen Inc.). Gel-purified PCR products were sequenced and analyzed as previously described.

**Table 2:** *PAX6* primer sequences used for exon scanning.

Exon	Primer (location)	Primer sequence	Tm (°C)	Product size (bp)
1	PX6-40F (5'UTR)	TCAGGCGCAGGAGGAAGTG	59.9	194
	PX6-41R (intron 1)	TCAGCGGCTGGAGAGTGAG	59.3	
2	PX6-42F (intron 1)	CTCACTCTCCAGCCGCTGAC	60.1	400
	PX6-43R (intron 2	CTCTCCCGGCGTGGCAGTG	63.6	
3	PX6-44F (intron 2)	GAGAGTCCATGGGCCTGTGC	60.6	200
	PX6-45R (intron 3)	CTTCTCCCATGTGAACAATGAAG	53.9	
4	PX6-84F (intron 3)	GCGACTGAGTGGATCCCTTC	58.2	302
	PX6-83R (intron 4)	CCCTCCAGCCGGACTGC	61	
5	PX6-86F (intron 4)	CCGCATGGACGTGTGGTCC	61.7	558
	PX6-79R (intron 5)	GGAGTTATCTTATGTGACTGAC	51.4	
5A	PX6-50F (intron 5)	CTCTCTACAGTAAGTTCTCATAC	49.6	210
	PX6-39R (exon 6)	CCAGTCTCGTAATACCTGCC	53.7	
6	PAX6-34F (exon 5A)	GTCCAAGTGCTGGACAATC	51	917
	PAX6-29R (intron 6)	AACAAGCTCCCAGCCATCCT	53.7	
7	PX6-51F (intron 6)	GTTGTTCTTTAAGAGAGTGGGTG	53.5	310
	PX6-52R (intron 7)	CCAGTGGCTGCCTATATGGAG	57.3	
8	PX6-53F (intron 7)	GCCTCTTTGGGAGGCTCCAAG	60.4	300
	PX6-54R (intron 8)	CTGGCTAAATATAGCTCTTTGTAC	51.2	
9	PAX6-30F (intron 8)	GCCACATCTTCAGTACAAAG	49.6	800
	PX6-21R (exon 10)	GCCTGTCTTCTCTGGTTCC	53.1	
10	PAX6-6F (exon 9)	GATCTACCTGAAGCAAGAATACAGG	54.9	590
	PX6-23R (exon 11)	GTGTTTGTGAGGGCTGTGTC	53.7	
11	PX6-22F (exon 10)	CCCAGTCACATCCCCATCAG	55.8	350
	PX6-55R (intron 11)	CCCACTCCTCGCTTCTCCG	60.1	
12	PX6-61F (intron 11)	CGATCATCAGGCTATCATAC	49.9	415
	PX6-62R (intron 12)	CCAGGAGGTTTCTCTTAAGG	52.2	
13	PX6-63F (intron 12)	CATAGTCCATGCTTGTCTCTC	52.7	318
	PX6-88R (3'UTR)	CACAGGACACAACTGCAGAAC	57.4	

### Radiation Hybrid Mapping

Canine *PAX6* was positioned on a radiation hybrid map using a canine/hamster radiation hybrid (RH3000) panel consisting of 92 cell lines made by fusing 3000 rad-irradiated canine fibroblast cells with TK-HTK3 hamster cells (Research Genetics, Inc., Huntsville, AL). Canine *PAX6* was linked to CFA18 in relation to 7 gene markers (*WT1*, *CD44*, *COLF1*, *COLF2*, *DLA79*, *ROM1*, and *TPCR63*) and 7 microsatellite markers (Wilms-TF, REN47J11, REN248C19, C18.156, C18.460, AHT130, and FH2356). All of these markers were previously mapped and positioned on CFA18 [21,22]. Primers for RH mapping are listed in Table 3. *PAX6* primers 30F and 21R, which amplify intron 8, were used to amplify *PAX6* in the RH3000 panel.

**Table 3:** Primers used for radiation hybrid mapping.

PRIMER	PRIMER SEQUENCE	Tm (°C)	PRODUCT SIZE (bp)
PAX6-30F (intron 8)	GCCACATCTTCAGTACAAAG	49.6	300
PAX6-31R (intron S)	TAGTTCAGGCATTGACTGATG	50.3	
1 WT1 (Wilms tumor 1)-F	GGTGCCTGGAAACGTCCG	59.2	155
WT1 (Wilms rumor 1)-R	ACCGGGAGAACTTTCGCTGAC	59 5	
1 CD44 (Cell differentiation antigen 44 variant)-F	TGGAAGAGAAGGTGGACATCTTCC	58.2	106
CD44 (Cell differentiation antigen 44 variant)-R	GGTCACCGGGATGAGGGTC	60.1	
COLF1 (Canine olfactory receptor gene 1) -F	GTCTCGGGGCATCTGTGTAT	57.4	357
COLF1 (Canine olfactory receptor gene 1) -R	GATGGCCACAGAAGTCAGGT	57.4	
COLF2 (Canine olfactorv receptor sene 2)-F	AGAGTGTGCTCCCTGCTGAT	57.4	357
COLF2 (Canine olfactory receptor sene 2)-R	TGCAACAGCAGTTAAGTGGG	55.4	
DLA79 MHC class IB) -F	TCTATTCTGGCATTGGGGAC	55.4	270
DLA79 (MHC class IB) -R	TGAGTAGCTCCCTCCTTTTCTG	58.1	
ROM1 -F	CTCTTTGATCCTCGTCAGCC	57.4	230
ROM1 -R	TGAGGGTCAGTAGGTCCCTG	59.5	
TPCR63 (Putative olfactory receptor)-F	AGGATACGTTCCTCAGAGGGCC	61.9	129
TPCR63 (Putative olfactory receptor)-R	ATCTAATGAGTGGTTGGTCCCTGGT	60.6	
Wilms-TF (tetra repeat)-F	CCCAATCTCCAGAGATTTTCC	52.3	300
Wilms-TF (tetra repeat)-R	CCAGTCTCAGCTGTGTCCAA	53.7	
REN47J11-F (CA)ll	TCTCCTCGCGTGTTTCTG	50.2	163
REN47J11-R (CA)ll	GGGGACACTCAGAAGGACG	55.3	
REX24SC19-F(CA)10	TGACTGTGGCAAGCAAGAAC	51.7	319
REX24SC19-R (CA)10	GGCAAAGAAAGATGGACTGG	51.7	
C1S.156-F(AC)15	ACAACCAACACACACAAAAACC	54.7	136
C18.156-R(AC)15	TGTTATCCCAGTGGCATTAGG	54.5	
C18.460-F (TG)17	CTTCCCATTATAGCCCTGTCC	54.8	147
C18.460-R (TG)17	GGTGTCAGG AAAATGAGACCA	548	
AHT130-F (GT)19	CCTCTCCTGGTAAGTGCTGC	57.6	113
AHT130-R (GT)19	TGGAACACTGGTCCCCAG	57.0	
FH2356-F (tetra repeat)	CTTGCATTCCCGCTCTCACT	57.6	235
FH2356-R (tetra repeat)	TCCTGAAATAGCTCCAGCGC	57.4	

PCR reactions were run on the 92 cell lines of the RH3000 panel, in duplicate or triplicate, at 25 μl volume. PCR conditions were as follows: 2 min at 94 °C, followed by 30 cycles of 30 s at 94 °C, 30 s at 56 °C, 30 s at 72 °C, and a final extension of 5 min at 72 °C. PCRs were optimized by adjusting the annealing temperature as needed. PCR products were electrophoresed and analyzed as described earlier. Gel images were recorded for each gel and scored for presence or absence of the band of interest in each of the 92 cell lines. Radiation hybrid data was analyzed using Multimap® software [23].

### Association Testing

**Figure 3:**
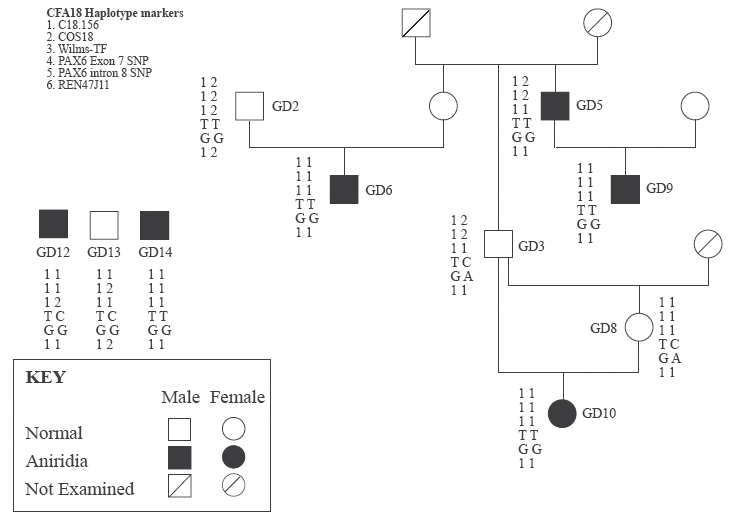
Test for association of aniridia phenotypes with CFA18 markers. Four microsatellites and 2 single nucleotide polymorphisms were analyzed in 10 dogs (6 affected, and 4 non-affected). Two of the 6 affected dogs were heterozygous; one of these was hetrozygous for the *PAX6* exon 7 SNP.

Ten dogs, 6 affected and 4 non-affected, were used to test for association of aniridia with the *PAX6* locus. Seven of the ten animals were part of one small pedigree and 3 were unrelated (Figure 3). Autosomal recessive inheritance was assumed based on a previous report [18], and limited pedigree information, but phenotypic variability, prevented the exclusion of dominant inheritance with incomplete penetrance. In addition, a founder effect mutation was hypothesized based on the small breed size. This mutation appears to have occurred recently in that the previous clinical [18] report is the first and only description of aniridia in the breed, and, as this is a working dog breed, the visual impairment caused by aniridia would be readily recognized in these dogs once it occurred.

Four microsatellite markers (C18.156, COS18, Wilms-TF, and REN47J11) which were previously mapped to CFA18 using an RH5000 panel [21,22] were used for association testing along with 2 SNPs identified within canine *PAX6*: one in exon 7 and one in intron 8. In exon 7 there is a T>C change (ACT>ACC) at nucleotide 501 of the 1,311 bp coding sequence (PAX6(5a)) that does not alter the translated amino acid (threonine). In intron 8 (which is 484 bp), there is a G>A change at intronic nucleotide position 351.

Primers used for association testing, along with PCR product sizes, genomic locations, and inter-marker distances are listed in Table 4. The previous RH5000 map placement of the 4 microsatellite markers gives 22.0 cRays between C18.156 and COS18, 57.5 cRays between COS18 and Wilms-TF, and 64.7 cRays between Wilms-TF and REN47J11 [22]. According to the current genomic sequence, *PAX6* lies between Wilms-TF and REN47J11 (Table 4). PCRs were run as previously described, and products for microsatellite markers were electrophoresed on 6% polyacrylamide gels, and microsatellite sizes were scored for each animal. PCR products for the exon 7 SNP were electrophoresed on 2% agarose gels (Invitrogen), gel purified, sequenced, and analyzed as previously described. Intron 8 PCR products were digested with *Bst*NI (New England Biolabs, Ipswich, MA) and electrophoresed on 6% polyacrylamide gels. Dogs homozygous (G/G) for the intron 8 SNP had 230 bp and 220 bp bands, while heterozygous dogs (G/A) had 230 bp, 220 bp, and 450 bp bands after enzyme digestion. Homozygous (A/A) dogs would have a 450 bp band, but there were none in this pedigree.

**Table 4:** Primer sequences used for association testing and genomic location of amplicons.

Primer name	Primer sequence	Tm (°C)	Product size (bp)	Canine genomic sequence location	Distance between markers
C18.156-F (AC)15	ACAACCAACACACACAAAAACC	54.7	136	Chr18:25401870+25402003	
C18.156-R (AC)15	TGTTATCCCAGTGGCATTAGG	54.5			2.2 Mb
					
COS-18-F (TC)19	CGTGGTGCCGGCCCTTTGAT	63.4	360	Chr18:27577535-30076377*	
COS-18-R (TC)19	TTTAGCGCCTGCCTTTGGAC	58.4			8.1 Mb
					
Wilms-TF-F (tetra repeat)	CCCAATCTCCAGAGATTTTCC	52.3	300	Chr18:38163822+38164112	
Wilms-TF-R (tetra repeat)	CCAGTCTCAGCTGTGTCCAA	53.7			510 Kb
					
PAX6-51F (exon 7 SNP)	GTTGTTCTTTAAGAGAGTGGGTG	53.5	310	Chr18:38675041-38687464**	
PAX6-52R (exon 7 SNP)	CCAGTGGCTGCCTATATGGAG	57.3			2.8 Kb
					
PAX6-30F (intron 8 SNP)	GCCACATCTTCAGTACAAAG	49.6	824	Chr18:38687699+38688522	
PAX6-21R (intron 8 SNP)	GCCTGTCTTCTCTGGTTCC	53.1			6.6 Mb
					
REN47J11-F (CA)11	TCTCCTCGCGTGTTTCTG	50.2	163	Chr18:45329434-45329603	
REN47J11-R (CA)11	GGGGACACTCAGAAGGACG	55.3			

The exact location of COS18* and *PAX6* exon 7** could not be determined in the current canine genome draft sequence. The site given for *COS18* represents the region between its flanking markers BAC_375-H17 and BAC_373-K16 [24,25]. The site given for exon 7 represents the last sequence before a gap in the genome which occurs in intron 4, and the calculated location of the beginning of exon 8, which is also located within the gap.

### Southern blot

Southern (DNA) analysis was performed using 10 μg of gDNA from two non-affected dogs (GD2 and GD8), two affected progeny (GD6 and GD10), and one control dog (beagle). Genomic DNA was digested with restriction enzyme *Eco*R1 (New England Biolabs, Ipswich, MA), electrophoresed on a 0.8% agarose gel, and transferred to a nylon hybridization transfer membrane (GeneScreen Plus® Perkin Elmer, Boston, MA). The nylon membrane was prehybridized and hybridized with ExpressHyb^TM^ (Clontech, Palo Alto, CA). Probes were labeled with a P^32^dCTP (3,000 Ci/mmol, 10 mCi/ml) using the RadPrime DNA Labeling System (Life Technologies^TM^, Inc Palo Alto, CA).

Two probes were designed from the canine *PAX6*. A 580 bp fragment, including all of exon 1, intron 1, exon 2, and part of intron 2, was used as a 5'UTR probe. This probe was amplified using primers *PAX6*-40F and 43R (Table 2). A 373 bp fragment in the PST domain, including part of intron 11, all of exon 12 and part of intron 12, was used as a 3' end probe. This probe was amplified using *PAX6*-61F and 62R (Table 2). Each probe was amplified in gDNA, gel purified using the QIAquick® Gel Extraction kit (QiagenIAGEN Inc.), and verified by sequencing.

## Results

### Characterization of canine *PAX6*

**Figure 4:**
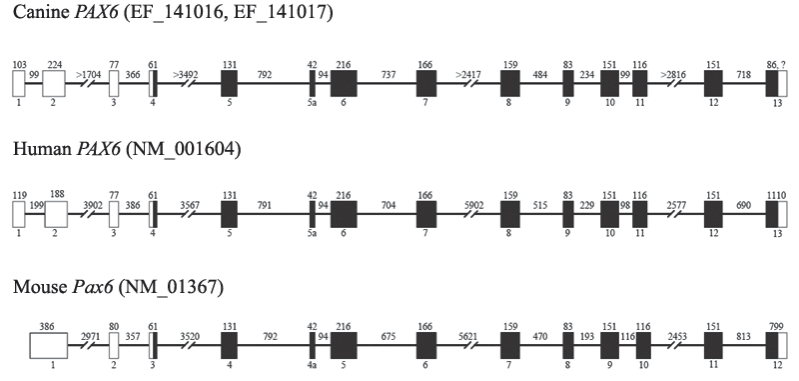
Comparison of canine, human and mouse *PAX6* organization. Open boxes represent non-coding exons, and closed boxes represent coding exons. Exon sizes are given above exon boxes. Lines represent introns with sizes written above.

A 1,072 bp fragment of canine *PAX6* cDNA, representing the 3' end of exon 4 through the 5' end of exon 12, was amplified from a canine retinal cDNA library using primers designed from human/mouse *PAX6* consensus sequence (Table 1). cDNA library vector primers, PBKIII and PBKVI, were used to extend the sequence to 1,472 bp which included the 3' end of exon 2 through the 5' end of exon 13. This fragment contained the complete *PAX6* coding sequence for isoform a, which does not contain the alternatively transcribed exon 5a. This 1,269 bp coding sequence began at the ATG start codon in exon 4, and ended at the TAA stop codon in exon 13. A poly-A sequence of at least 20 residues followed the stop codon, and it was unclear where non-coding exon 13 ended. Similarly, in man the ATG start codon occurs in exon 4, and the TAA stop codon in exon 13 is followed by a poly-A sequence of 21 residues. Exon 13 in man is estimated to be 1,110 bp (NM_001604). Unlike the human and canine genes, mouse *Pax6* contains 12 exons instead of 13, and mouse exons 2-12 correspond with human and canine exons 3-13 (Figure 4). As a result, the mouse ATG start codon is in exon 3 (instead of exon 4), and the TAA stop codon is in exon 12 (instead of exon 13), and it is not followed by a poly-A sequence (NM_013627).

A 42 bp alternatively transcribed exon 5a was amplified from the canine retinal cDNA library, and was located between exons 5 and 6, as in man. This alternatively transcribed exon is located between exons 4 and 5 in the mouse. The coding sequence for canine *PAX6* isoform b, which includes this alternatively transcribed exon, was 1,311 bp, and was the same length as the human and mouse *PAX6*(5a) coding sequences.

Sequences for exon 1 and the 5' end of exon 2 could not be amplified from the cDNA library, and were obtained from the TIGR 1.5X (poodle) canine genomic sequence [20]. Within the 5' UTR, the predicted sizes for canine exons 1 and 2, and introns 1 and 2, were similar in size to the corresponding human exons and introns (Table 5). Exon 1 was predicted to be 103 bp based on the TIGR canine genomic sequence, and sequences amplified from genomic DNA in canine patients. In man, *PAX6* exon 1 is 119 bp (NM_001604) or 196 bp (NM_000280). *PAX6* exon 2 is 188 bp in man (NM_001604 and NM_000280), and predicted to be 224 bp in the dog. The additional 36 bp in canine exon 2 included a 23 bp poly-A sequence near the center of the exon followed by highly repetitive sequence. In the mouse, the comparable exon 1 is 386 bp (NM_013627), while exon 2 is 80 bp and corresponds with human and canine exon 3 (77 bp; Figure 4 and Table 5).

**Table 5:** Comparison of human, canine and mouse *PAX6* exon and intron sizes.

***PAX6* EXONS & INTRONS**	HUMAN* SIZES (bp)	**CANINE SIZES (bp)**	**MOUSE EXONS/ INTRONS**	MOUSE** SIZES (bp)
EXON 1	119	103		
INTRON 1	197	99		
EXON2	188	224	EXON 1	386
INTRON 2	3902	≥1704^†^	INTRON 1	2971
EXON3	77	77	EXON 2	80
INTRON 3	386	366	INTRON 2	357
EXON 4 (ATG)	61	61	EXON 3 (ATG)	61
INTRON 4	3567	≥3492^Δ^	INTRON 3	3520
EXON 5	131	131	EXON 4	131
INTRON 5	791	792	INTRON 4	792
EXON 5A	42	42	EXON 4a	42
INTRON 5A	94	94	INTRON 4a	94
EXON 6	216	216	EXON 5	216
INTRON 6	704	737	INTRON 5	675
EXON 7	166	166	EXON 6	166
INTRON 7	5902	≥2417^†^	INTRON 6	5621
EXON 8	159	159	EXON 7	159
INTRON 8	515	484	INTRON 7	470
EXON 9	83	83	EXON 8	83
INTRON 9	229	234	INTRON 8	193
EXON 10	151	151	EXON 9	151
INTRON 10	98	99	INTRON 9	116
EXON 11	116	116	EXON 10	116
INTRON 11	2577	≥2816^Δ^	INTRON 10	2453
EXON 12	151	151	EXON 11	151
INTRON 12	690	716	INTRON 11	813
EXON 13 (TAA)	1110	>99	EXON 12 (TAA)	799

The asterisk indicates that human sizes were determined using reference sequence NM_001604 and the UCSC human genome sequence. The double asterisk indicates that mouse sizes were determined using reference sequence NM_013627 and the UCSC mouse genome sequence. The "cross" indicates that canine intron 2 and 7 estimates are based on the TIGR 1.5X (poodle) sequence. ΔCanine intron 4 and 11 estimates are based on UCSC 7.5X (boxer) sequence. The complete size of canine exon 13 is unknown. The ATG start codon is located in human and canine exon 4, and mouse exon 3. The TAA stop codon is located in human and canine exon 13, and mouse exon 12.

The canine *PAX6* cDNA sequence (1,786 bp) representing exons 1-13, including exon 5a, and a 20 bp poly-A 3' end, was analyzed against the 2005 canine 7.6X genomic sequence (boxer) using the blat function. As of November, 2006, there was 99.9% identity spanning >21 kb in the genomic sequence (38,671,042-38,692,610) on chromosome 18. Matching sequences were identified for exons 3-4, and 9-13. However, *PAX6* exons 1-2 and 5-8 (including exon 5a) were missing from the current canine draft sequence. A 714 bp gap in the canine sequence, from 38,666,389-38,667,102, included predicted exons 1 and 2, while a second, larger 12,622 bp gap from 38,675,042-38,687,663, included exons 5-8. As a result, our cloned sequence provides the complete *PAX6* coding sequence (accession numbers EF141016, and EF141017) which was unavailable from the latest canine genomic sequence.

**Figure 5:**
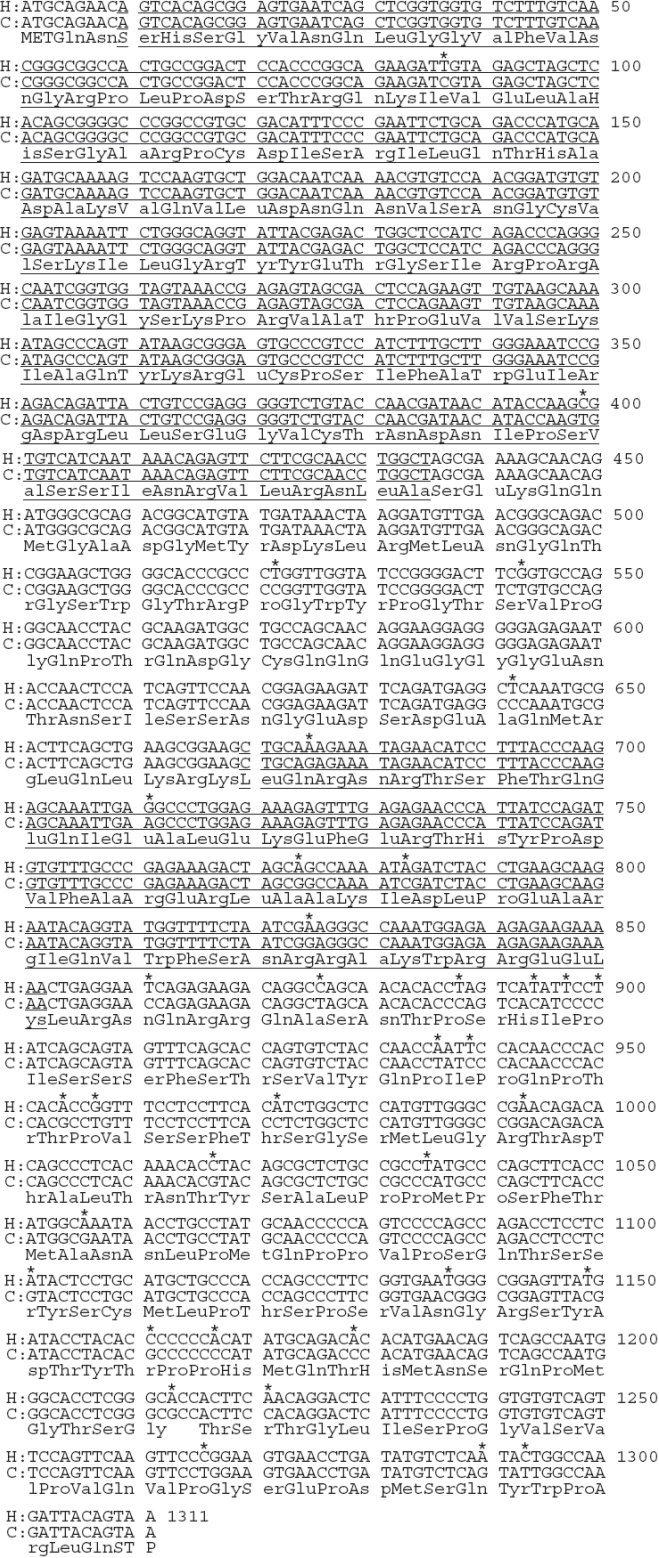
Comparison of the canine and human *PAX6* coding sequence (CDS), and amino acid sequence. Human (NM_001604) CDS (first row); canine (EF141016) CDS (second row); corresponding amino acid sequence (436 aa; third row). The first underlined region is the paired domain (426 bp), followed by the linker region (234 bp). The second underlined region is the homeodomain (183 bp), followed by the PST domain (459 bp). The asterisks mark the 36 nucleotide differences between dog and man, two in the paired domain; three in the linker; five in the homeodomain; and 26 in the PST domain. Thirty-five of the 36 nucleotide differences occurred in the third position of the triplet codon, and did not alter the translated amino acid. Only one nucleotide difference occurred at the first position of a codon (base pair 1,213) resulting in an ACC (threonine) in man, and a GCC (alanine) in the dog. This change occurred in exon 12 which was part of the PST domain. As a result, the human and canine *PAX6* nucleotide coding sequences were 97.3% identical, and the corresponding amino acid sequences were 99.8% identical.

Primers designed to cross introns were used to determine intronic sizes for 9 of the 13 canine introns. The estimated sizes of the remaining 4 introns (2, 4, 7, and 11) were determined using genomic sequence information from TIGR 1.5X (poodle) [20] and the 7.6X (boxer) [17] public canine genome sequence (Figure 4). Canine exons 3-12, and intron 5a are identical in size to the human counterparts, while exons 1, and 2, and introns 1-12 are similar in size (Table 5). Both canine and human isoform b (*PAX6[5a]*) nucleotide coding sequences were 1,311 bp and shared 97.3% identity, while the corresponding amino acid sequences shared 99.8% identity (Figure 5). Interestingly, the canine and mouse coding sequences were only 93.2% identical, and had 89 nucleotide differences: 15-PD, 13-Linker, 11-HD, 50-PST. Eighty-four of the 89 nucleotide differences between dog and mouse occurred in the 3rd codon position and represented silent changes. Of the 5 nucleotide differences which occurred in the first codon position, only two resulted in an amino acid change: *PAX6(5a)* coding sequence nucleotide position 178 in exon 5a (PD) coded for glutamine (CAA) in the dog and man, but glutamic acid (GAA) in mouse; nucleotide position 1,213 in exon 12 (PST) coded for alanine (GCC) in the dog and threonine (ACC) in mouse and man.

### Exon scanning

The 14 exons of canine *PAX6* were scanned for mutations, including predicted exons 1 and 2, cloned exons 3-13, and the alternatively transcribed exon 5a. Splice sites at the intron/exon boundaries were also examined; at least 27 intronic nucleotides before each exon, and at least 30 intronic nucleotides after each exon were included in the analysis. *PAX6* exon scans were performed in 7 dogs that were part of one pedigree; this included 4 aniridia-affected dogs and 3 non-affected related dogs (Figure 2). Non-coding exon 2 (224 bp) contained a 23 bp poly-A region followed by 6 AACC repeats. The first 133 bp of exon 2 was scanned in all 7 dogs, and the complete exon 2 sequence was scanned in 2 affected dogs (GD 5, 6) and 2 normal dogs (GD 3, 8). These 4 dogs had an extra AACC repeat in non-coding exon 2.

*PAX6* exon scanning also revealed 2 single nucleotide polymorphisms (SNPs). The first was located in exon 7 at nucleotide position 501 (within the 1311 bp coding sequence), and involved a C>T change at the third position of the codon which did not alter the amino acid coded (ACC to ACT; threonine). The second SNP occurred in intron 8 (484 bp) at intronic nucleotide position 351, and was an A>G transition. Both of these SNPs were silent changes which did not alter the translated amino acids. No pathological mutations were identified in all 14 exons scanned or in their exon/intron junctions.

### Radiation hybrid mapping

A canine/hamster radiation hybrid (RH3000) panel was used to link canine *PAX6* to CFA 18 in relation to 7 gene markers and 7 microsatellite markers (Table 3) which were mapped in previous studies [21,22]. Multimap® analysis could only place 8 of the 15 markers at unique map positions. *PAX6* and 6 other markers were placed on CFA18, but could not be localized to a unique position in relation to the other markers. However, two-point linkage analysis linked canine *PAX6* to Wilms-TF and WT1 on CFA18, with LOD scores of 8.0 and 7.1, respectively. Wilms-TF and WT1 are both located on CFA18 in a region with homology to HSA11p13 [22,24,25],  which is where human *PAX6* is located. Similar map locations between the canine and human *PAX6* genes further supports their homology.

### Association testing

Having excluded disease-associated changes in the coding sequence, we examined whether there was an association of *PAX6* with the disease locus. Ten Catalan sheepdogs (6 affected and 4 non-affected) were tested for association of *PAX6* with the aniridia phenotype. Seven of these dogs were related and part of the same pedigree, while 3 dogs were unrelated (Figure 3). Because of the limited genetic studies to date, it was not definitive whether this disease was recessive or dominant. Based on a prior report [18], and the production of affected dogs from non-affected parents, recessive inheritance was assumed. Furthermore, based on the small breed size, we hypothesized also that all aniridia-affected dogs resulted from a founder-effect mutation. Thus, if the disease is autosomal recessive, all affected dogs would be homozygous for a common haplotype segment of CFA18. Alternatively, if the disease is autosomal dominant with incomplete penetrance, all affected dogs would have at least one common haplotype segment of CFA18.

Six polymorphic markers on CFA18 were analyzed that included 4 microsatellite markers (C18.156, COS18, Wilms-TF, and REN47J11), and 2 *PAX6* SNPs (exon 7 and intron 8). All 10 dogs examined shared at least one common haplotype for all 6 markers (Figure 3; "111TG1"). Four of the 6 affected dogs (GD6, GD9, GD10, and GD14) were homozygous for this common haplotype, while 2 affected dogs (GD5 and GD12) were heterozygous. All affected dogs, regardless of homozygosity or heterozygosity for this haplotype, showed a broadly similar aniridia phenotype. Haplotypes for GD5 and GD12 were repeated to confirm their heterozygosity. All 4 unaffected dogs (GD2, GD3, GD8, and GD13) were heterozygous for the common haplotype. At 4 of the 6 individual marker loci (C18.156, COS18, WILMS-TF, and exon 7 SNP) both normal and affected dogs shared common genotypes. For 2 marker loci (intron 8 SNP and REN47J11) non-affected dogs had both homozygous and heterozygous genotypes while all affected dogs were homozygous. Furthermore, at each marker locus there were both affected and non-affected animals with identical genotypes. Such degree of haplotype sharing is not unexpected given the small breed size and degree of inbreeding.

Assuming recessive inheritance, our observations suggested no association of the *PAX6* locus with the aniridia phenotype. However, if aniridia segregates as a dominant disease in these dogs, our observations would suggest an association in that all affected dogs shared at least one common haplotype ("111TG1"). Incomplete penetrance could explain the presence of this commom haplotype in "non-affected" dogs (GD2, GD3, GD8, and GD13) that did not manifest the aniridia phenotype.

### Southern blot analysis

A Southern blot analysis was performed using probes designed from both the 5' UTR (exon 1, intron 1, exon 2, and the 5' end of intron 2) and the 3' PST domain (3' end of intron 11, all of exon 12, and the 5' end of intron 12) of *PAX6*. Identical bands were found in affected and non-affected dogs from the aniridia pedigree, and a normal beagle control using both probes. The 5' UTR probe hybridized at about 8,500 bp, while the 3' PST probe hybridized at about 7,400 bp (data not shown). The absence of any distinction between the *PAX6* bands of affected and non-affected animals using both a 5' and a 3' end probe suggested that there were no large deletions present in the *PAX6* gene.

## Discussion

Human aniridia (OMIM 106210) patients show varying degrees of iris loss including complete to partial absence of the iris, iris coloboma, and thinning of the iris. They may also exhibit other related ocular abnormalities including cataract, keratitis, or glaucoma. In this study, the clinical manifestation of aniridia in a canine model strongly resembled that seen in human patients. Canine subjects showed total to nearly complete absence of the iris with ciliary processes visible at the circumferential border; in some cases, cataracts, corneal edema, and glaucoma also were present. The anatomical similarities between the dog and human eye, along with the clinical similarities in canine and human aniridia phenotypes, make the dog an important model for this disease.

Because the vast majority of aniridia cases in man are caused by mutations in the *PAX6* gene [26] we used the candidate gene approach to evaluate *PAX6* in the study population. *PAX6* was cloned from a canine retinal cDNA library, and linked to WT1 and Wilms-TF on CFA18 using an RH3000 panel. A previous study placed *PAX6* on CFA18 in the same region between markers FH3824 and CFOR04B04 using an RH5000 panel [27]. WT1 is just 4 cRays away from marker FH3824, and 14 cRays from CFOR04B04 on the RH5000, 4249-marker map [25]. Integrated canine maps demonstrate homology between CFA18 and human chromosomes 7 and 11 [28-30]. *PAX6* is located in a region of CFA18 which has homology with human chromosome 11p13, where *PAX6* is located in man.

The canine *PAX6* cDNA sequence was 1,786 bp, and included predicted exons 1 and 2, and cloned exons 3-13, plus alternatively transcribed exon 5a (EF141016, EF141017). This cDNA has sequence identity with the current canine genomic sequence over a span >21 kb in length (38,671,042-38,692,610) [17]. This presumably represents the genomic length of the canine *PAX6* gene. The human *PAX6* genomic sequence is 22.4kb (NM_001604), while the mouse genomic sequence is 20.6kb (NM_013627). Canine *PAX6* exons 3-4 and 9-13 matched sequences in the current genomic sequence. However, exons 1-2 fell within a 714 bp gap, and exons 5-8 fell within a second 12,622 bp gap in the sequence. The exons that are missing in the current draft canine genomic sequence are provided by our cloning studies. Thus, this study fills in the gaps left by the draft canine genome sequence, and provides a complete canine *PAX6* sequence.

At the nucleotide level, the canine *PAX6(5a)* coding sequence (1,311 bp) had a higher degree of homology to the human (NM_001604; 97.3% identical) than to the mouse (NM_013627; 93.2% identical) sequence. Most of the nucleotide variations occurred within the PST domain: 26 of the 36 nucleotide differences between dog and human, and 50 of 89 differences between dog and mouse. Nevertheless, most of the nucleotide differences occurred in the third codon position and were synonymous changes which did not result in corresponding amino acid changes (Figure 5).

At the amino acid level, canine PAX6 is 99.8% and 99.5% identical, respectively, to the human and murine sequences. The one amino acid difference between canine and human sequences occurred in the PST region at codon 405 (nucleotide position 1213, within the 1311 bp coding sequence) which codes for alanine in the dog, and threonine in man. The mouse also has threonine at codon 405, and, in addition, the mouse has glutamic acid in the PD at codon 60 (nucleotide position 178, within the 1311 bp coding sequence) while the dog and human have glutamine. The extremely high *PAX6* nucleotide and amino acid sequence identities between dog, man, and mouse suggest a consistency in protein structure and function.

Both canine (EF141016, EF141017) and human (NM_001604) *PAX6* contained 13 exons plus an alternatively transcribed exon 5a. Exons 3-12 and intron 5a were the same sizes in both species while intronic sizes were similar. The mouse (NM_013627) gene, however, only has 12 exons (plus exon 4a) with mouse exons 2-12 corresponding to canine and human exons 3-13. The mouse coding sequence (exons 3-12) was the same size as the corresponding coding sequences in dog and human (exons 4-13). Mouse exon 2, and introns 1-12 were also similar in size to canine and human, but mouse exon 1 was 386 bp compared with 103 bp (dog) and 119 bp (human). Mouse exon 1 is more similar in size to canine and human exons 1 and 2 combined, 327 (canine) and 307 (human), and may explain why the mouse has one less exon than the canine and human genes.

No pathological mutations were detected in the canine *PAX6* coding sequence, non-coding exons, or in the intron/exon junctions of aniridia-affected dogs. This included coding exons 4-13, the alternatively transcribed exon 5a, and the non-coding exons 1-3 in the 5' UTR. Because the dogs used in this study were privately owned pets or working dogs, we were unable to obtain tissue samples to examine *PAX6* RNA products to detect splicing defects or other transcript variants. We also were unable to examine *PAX6* expression in normal and affected animals to rule out mutations in a promoter or enhancer.

Limitations in access to a larger sample size of dogs, or the ability to carry out prospective matings, prevented confirmation of the mode of inheritance of canine aniridia, and created difficulties in interpreting the association test. If canine aniridia is inherited as a recessive trait, the association test suggests no association of the *PAX6* locus with the aniridia phenotype. However, if canine aniridia is a dominant disease, there appears to be association of *PAX6* with aniridia in that all affected dogs share at least one common haplotype ("111TG1"). Dominance with incomplete penetrance could account for the presence of this common haplotype in the "non-affected" dogs (GD2, GD3, GD8, and GD13). Nonetheless, the broadly similar aniridia phenotype present in dogs homozygous or heterozygous for the "111TG1" haplotype argues against dominant inheritance. This emphasizes the need for further analysis of animals to establish the mode of inheritance, and to conduct a more complete association test and segregation analysis.

Regardless of the mode of inheritance, the Southern blot analysis showed no difference in normal from affected dogs using both a 5' and a 3' end probe which ruled out a large deletion in the *PAX6* gene. In the case of a recessively inherited deletion, normal and affected dogs would be distinguishable on Southern analysis because all affected dogs would be homozygous and have either no *PAX6* band (if the probe is in the deletion), or a smaller *PAX6* band (if the probe is not in the deletion, and the deletion is greater than or equal to 50 bp). In the case of dominant inheritance, a large deletion in the *PAX6* gene may go undetected in a heterozygous-affected dog if the probe lies within the deletion. However, because we used both 5' and 3' end probes, we should detect a 5' end deletion with the 3' end probe and vise versa, unless the entire gene is deleted. We know that the entire gene is not deleted because of the heterozygosity detected in exon 7 (GD3, GD8, GD12, and GD13) and intron 8 (GD3 and GD8) SNPs. It is very unlikely that a genomic deletion that spares the exon 7 and intron 8 regions would also be unrecognizable using probes at both the 5' and 3' ends of the gene. As a result, we have excluded a large deletion in *PAX6* as a cause of aniridia in the Catalan sheep dog.
